# Impact of bridging thrombolysis versus endovascular thrombectomy alone on outcomes in anticoagulated patients with atrial fibrillation and acute ischaemic stroke

**DOI:** 10.1111/ene.16453

**Published:** 2024-08-23

**Authors:** Muath Alobaida, Stephanie L. Harrison, Deirdre A. Lane, Fiona Rowe, Philip Austin, Azmil H. Abdul‐Rahim, Gregory Y. H. Lip

**Affiliations:** ^1^ Liverpool Centre for Cardiovascular Science at University of Liverpool Liverpool John Moores University and Liverpool Heart & Chest Hospital Liverpool UK; ^2^ Department of Cardiovascular and Metabolic Medicine, Institute of Life Course and Medical Sciences University of Liverpool Liverpool UK; ^3^ Department of Basic Science, Prince Sultan Bin Abdulaziz College for Emergency Medical Services King Saud University Riyadh Saudi Arabia; ^4^ Registry of Senior Australians South Australian Health and Medical Research Institute Adelaide Australia; ^5^ Department of Clinical Medicine Aalborg University Aalborg Denmark; ^6^ Institute of Population Health University of Liverpool Liverpool UK; ^7^ TriNetX London UK; ^8^ Stroke Division, Department of Medicine for Older People, Whiston Hospital Mersey and West Lancashire Teaching Hospitals NHS Trust Rainhill UK

**Keywords:** anticoagulation, atrial fibrillation, bridging thrombolysis, endovascular thrombectomy, ischaemic stroke, outcome

## Abstract

**Background and purpose:**

The impact of bridging thrombolysis prior to endovascular thrombectomy (EVT) compared to EVT alone on intracerebral haemorrhage (ICH), subarachnoid haemorrhage (SAH), and death in anticoagulated atrial fibrillation (AF) patients with acute ischaemic stroke (AIS) is not well defined.

**Methods:**

A retrospective study was conducted using data from a federated research network (TriNetX) including 114 health care organisations in the United States. Anticoagulated AF patients with AIS who received either bridging thrombolysis (BT) or EVT alone from September 2018 to November 2023 were included. Following propensity score matching, Cox regression analyses examined the risk of ICH, SAH, and death within 30 and 90 days, comparing anticoagulated AF patients receiving BT versus EVT only.

**Results:**

A total of 3156 patients with AIS were treated with BT or EVT alone. Following 1:1 propensity score matching, the cohort included 766 patients in each group. ICH occurred within 30 and 90 days in 6.9% and 8.0% in the BT group compared with 7.4% and 7.7% in the EVT‐only group (hazard ratios [HR] = 0.92, 95% confidence interval [CI] = 0.63–1.33 and HR = 1.01, 95% CI = 0.71–1.45, respectively). SAH occurred within 30 and 90 days in 4.2% and 4.4% of patients in the BT compared to 3.0% and 3.4% in the EVT‐only group (HR = 1.38, 95% CI = 0.81–2.38 and HR = 1.29, 95% CI = 0.77–2.14, respectively). Death occurred within 30 and 90 days in 17.8% and 19.8% of patients in the BT compared to 22.2% and 27.3% in the EVT‐only group (HR = 0.77, 95% CI = 0.62–0.97 and HR = 0.65, 95% CI = 0.56–0.86, respectively).

**Conclusions:**

In anticoagulated AF patients with AIS, BT was associated with a significantly lower risk of death, with no difference in ICH or SAH risk within 30 and 90 days compared to EVT only.

## INTRODUCTION

Reperfusion therapies have been shown to improve clinical outcomes in patients with acute ischaemic stroke and large vessel occlusion (LVO) [1]. Bridging thrombolysis, comprising endovascular thrombectomy (EVT) plus intravenous thrombolysis (IVT), has proven major therapeutic effect and superiority over IVT alone in patients with acute ischaemic stroke and LVO [2]. A previous meta‐analysis of six randomised clinical trials has shown that bridging thrombolysis is not noninferior to EVT alone in terms of functional status, symptomatic intracerebral haemorrhage (sICH), and mortality [3]. Nevertheless, current evidence favours bridging thrombolysis in patients with ischaemic stroke and LVO (≤4.5 h of stroke onset) who are eligible for both treatments over EVT alone [4].

People with acute ischaemic stroke and atrial fibrillation (AF) often present with more severe strokes, as indicated by higher score on the National Institutes of Health Stroke Scale (NIHSS), and face increased risks of ICH and mortality compared to those without AF [5, 6]. Oral anticoagulant use may preclude AF patients from bridging thrombolysis when the international normalised ratio is >1.7 or if a direct oral anticoagulant (DOAC) has been administered within 48 h prior to stroke onset where specific coagulation testing is not available or results are unknown [7].

The evidence derived from post hoc analyses and observational studies that have assessed the efficacy and safety of bridging thrombolysis compared to EVT alone in anticoagulated patients with AF is limited [8, 9, 10]. Conflicting results have been reported in previous studies examining associations between AF and outcomes for anticoagulated patients with ischaemic stroke receiving bridging thrombolysis compared to EVT alone [10, 11, 12].

For example, a recent comprehensive systematic review and meta‐analysis reported limited studies examining the impact of bridging thrombolysis in patients with AF, and meta‐analyses examining the outcomes of ICH and 90‐day mortality could not be performed due to the scarcity of available studies [12].

Given the conflicting observations, the efficacy of bridging thrombolysis compared to EVT alone in anticoagulated patients with AF eligible for both treatments remains uncertain. We therefore assessed the risks of ICH, subarachnoid haemorrhage (SAH), and all‐cause mortality within 30 and 90 days in this context, using data from a large global federated health research network.

## METHODS

A retrospective observational study was conducted with data obtained from TriNetX (https://live.trinetx.com), which provides access to electronic medical records retrieved from 32 and 34 of 114 health care organisations, including specialised medical centres, academic and nonacademic medical centres, and community hospitals mainly in the United States. These data include patient demographics, diagnoses (using International Classification of Diseases, 10th Revision, Clinical Modification [ICD‐10] codes), medical procedures, medications, and laboratory measurements. This global federated network provides only anonymous data of electronic medical records with no patient‐identifiable information or identification of participating health care organisations; thus, research studies using this network do not require institutional review boards for ethical approval. The specific identities of these health care organisations are not disclosed, to ensure patient confidentiality and to comply with legal and ethical standards.

In this analysis, the TriNetX database was searched and patients meeting the following criteria were included: (i) entered on the register prior to 14 March 2024, (ii) aged 18 years or older, (iii) on oral anticoagulation and had an ICD‐10 diagnosis code of AF, (iv) ischaemic stroke from 1 September 2018 to 1 November 2023, (v) received IVT treatment (alteplase or tenecteplase), and (vi) received EVT treatment (extirpation or dilation procedure code). Patients in the EVT‐alone group met the same criteria as the bridging thrombolysis group but did not receive IVT treatment. The diagnostic and treatment codes used for the study are available in Table S1. The STROBE (Strengthening the Reporting of Observational Studies in Epidemiology) statement is available in Table S2. Thirty‐two and 34 of the 114 health care organisations in the TriNetX network within the study period had patients who met the study inclusion criteria for bridging thrombolysis and EVT‐alone cohorts, respectively. Given that the cohorts were predominantly derived from the United States, the start date of 1 September 2018 was chosen, because the American Heart Association/American Stroke Association guideline for early management of patients with acute ischaemic stroke was originally published in January 2019 [13], and the end date 1 November 2023 was chosen to allow for at least 90 days of follow‐up for all participants. The 30‐ and 90‐day outcome assessment has been recommended as the standard for all clinical and preclinical studies and trials intending to demonstrate the short‐term and long‐term benefit of acute treatment in stroke [14].

### Statistical analyses

Propensity score matching (nearest‐neighbour matching with a tolerance level of 0.01 and difference between propensity scores of ≤0.1) was used to control for differences in the comparison cohorts. A standardised mean difference (SMD) < 0.1 was considered well balanced, and propensity score density graphs were examined. Using logistic regression, patients with bridging thrombolysis were 1:1 propensity score matched to patients with EVT only for age, sex, race, hypertensive diseases, previous transient ischaemic attack, ischaemic heart disease, heart failure, hyperlipidaemia, diabetes mellitus, liver disease, chronic kidney disease, and NIHSS score. These variables were selected because they may have an effect on the clinical and prognostic outcomes. Following propensity score matching, Cox regression proportional hazard models were used to calculate hazard ratios (HRs) with 95% confidence intervals (CIs) for 30‐ and 90‐day incidence of ICH (subcortical, cortical, hemisphere, brain stem, cerebellum, intraventricular, multiple localised), SAH, and all‐cause mortality, comparing stroke patients with bridging thrombolysis with propensity‐matched controls (EVT only). Intracranial haemorrhages such as subdural haematoma were excluded. Kaplan–Meier survival curves for ICH, SAH, and all‐cause mortality at 30 and 90 days from the date of the index stroke by thrombolysis received were used. Statistical significance was set at *p* < 0.05. All analyses were performed on the TriNetX platform, which uses the R survival package v3.2‐3.

## RESULTS

**FIGURE 1:**
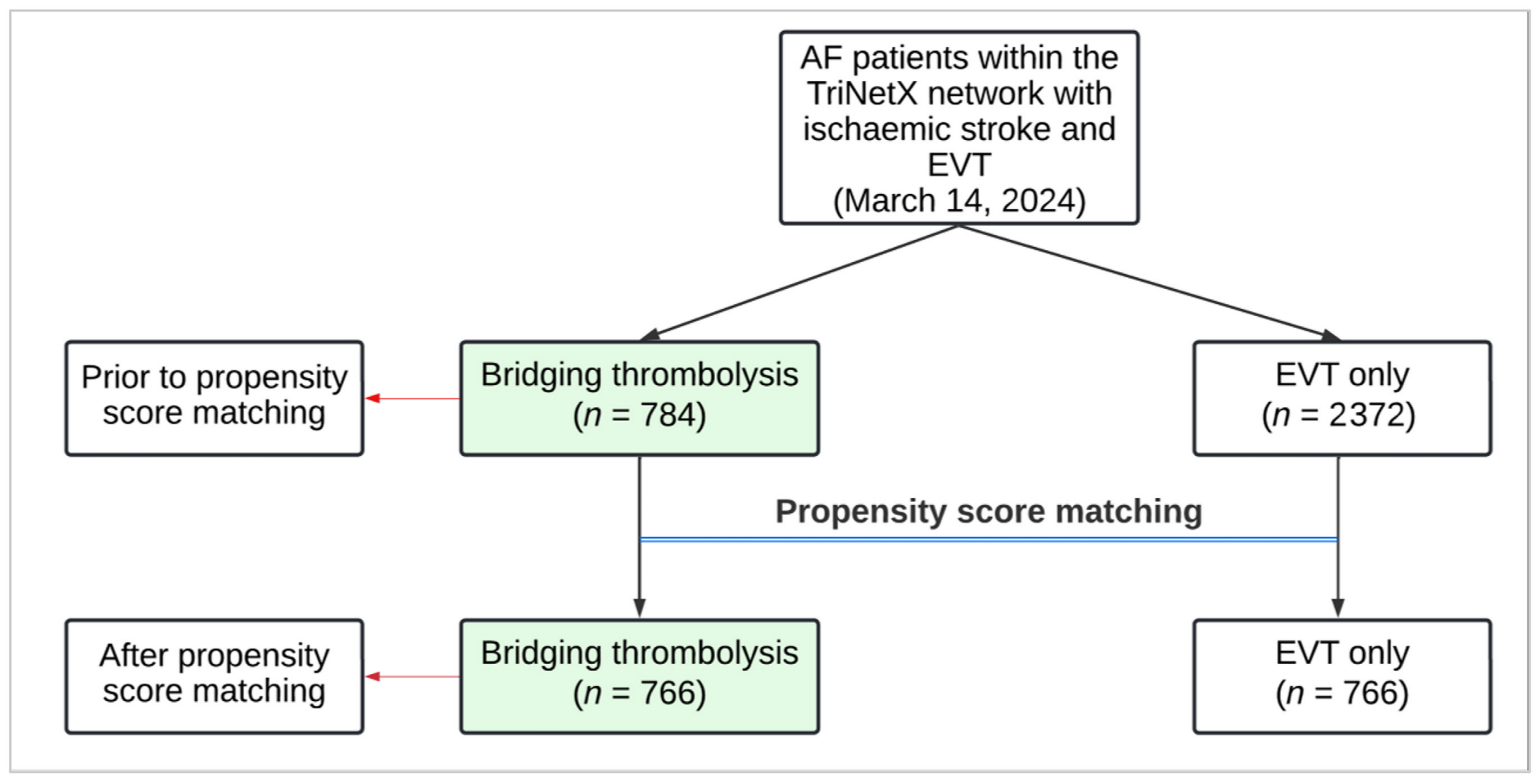
Flow diagram of selected patients with ischaemic stroke and atrial fibrillation (AF). EVT, endovascular thrombectomy.

Within the TriNetX network at the time of the analysis, there were 784 anticoagulated patients with ischaemic stroke and AF who received bridging thrombolysis, and 2372 anticoagulated patients with ischaemic stroke and AF who received EVT only between September 2018 and November 2023 (Figure 1). Patients who received bridging thrombolysis had a significantly higher prevalence of chronic obstructive pulmonary disease and hyperlipidaemia (Table 1) compared to those who received EVT only.

**TABLE 1:** Baseline characteristics for anticoagulated stroke patients with atrial fibrillation treated with bridging thrombolysis and controls treated with EVT alone, before and after propensity score matching.

	Before propensity score matching			After propensity score matching		
	Bridging thrombolysis, *n* = 784	EVT alone, *n* = 2372	SMD	Bridging thrombolysis, *n* = 766	EVT alone, *n* = 766	SMD
Demographics						
Age, years ± SD	75.3 ± 12.2	75.9 ± 11.2	0.06	75.3 ± 12.2	75.1 ± 11.1	0.02
Female, *n* (%)	399 (50.9)	1239 (52.2)	0.03	392 (51.2)	377 (49.2)	0.04
White, *n* (%)	490 (62.5)	1583 (66.7)	0.09	490 (64)	476 (62.1)	0.04
Black, *n* (%)	91 (11.6)	205 (8.6)	0.09	89 (11.6)	86 (11.2)	0.01
Asian, *n* (%)	79 (10.1)	141 (5.9)	0.15	70 (9.1)	84 (11)	0.06
Comorbidities						
Hypertension, *n* (%)	604 (77)	1757 (74.1)	0.07	590 (77)	591 (77.2)	0.003
Diabetes, *n* (%)	279 (35.6)	837 (35.3)	0.006	274 (35.8)	264 (34.5)	0.03
Previous TIA or ischaemic stroke, *n* (%)	176 (22.4)	597 (25.2)	0.06	175 (22.8)	189 (24.7)	0.04
Dyslipidaemia, *n* (%)	558 (71.2)	1533 (64.6)	0.14	544 (71)	546 (71.3)	0.006
Ischaemic heart disease, *n* (%)	338 (43.1)	988 (41.7)	0.03	329 (43)	325 (42.4)	0.01
Heart failure, *n* (%)	310 (39.5)	994 (41.9)	0.05	304 (39.7)	299 (39)	0.01
COPD, *n* (%)	99 (12.6)	386 (16.3)	0.10	98 (12.8)	102 (13.3)	0.02
Chronic kidney disease, *n* (%)	209 (26.7)	594 (25)	0.04	205 (26.8)	206 (26.9)	0.003
NIHSS, median (IQR)	17 (11–23)	16 (10–22)	0.05	17 (11–23)	17 (12–23)	0.004
Medication and laboratory parameters^a^						
Warfarin, *n* (%)	47 (6.0)	160 (6.7)	0.03	47 (6.1)	47 (6.1)	<0.001
INR, mean ± SD	1.2 ± 0.3	1.2 ± 0.4	0.15	1.2 ± 0.3	1.2 ± 0.5	0.09
PT, mean ± SD	14.4 ± 3.6	14.8 ± 4.3	0.12	14.4 ± 3.7	14.7 ± 4.4	0.08
Eptifibatide, *n* (%)	18 (2.3)	72 (3.0)	0.05	18 (2.3)	14 (1.8)	0.04
Enoxaparin, *n* (%)	147 (18.8)	413 (17.4)	0.03	142 (18.5)	149 (19.5)	0.02
Heparin, *n* (%)	645 (82.3)	1885 (79.5)	0.07	630 (82.2)	626 (81.7)	0.01
aPTT, mean ± SD	31 ± 9.1	31.8 ± 12	0.07	31 ± 8.9	31.9 ± 12.1	0.09
Dabigatran, *n* (%)	10 (1.3)	21 (0.9)	0.04	10 (1.3)	10 (1.3)	<0.001
Rivaroxaban, *n* (%)	39 (5)	128 (5.4)	0.02	39 (5.1)	40 (5.2)	0.006
Apixaban, *n* (%)	130 (16.6)	437 (18.4)	0.05	129 (16.8)	135 (17.6)	0.02
Statins, *n* (%)	513 (65.4)	1564 (65.9)	0.01	505 (65.9)	521 (68)	0.04

*Note*: Cohorts were considered well matched with a standardised mean difference between them of <0.1.

Abbreviations: aPTT, activated partial thromboplastin time; COPD, chronic obstructive pulmonary disease; EVT, endovascular thrombectomy; INR, international normalised ratio; IQR, interquartile range; NIHSS, National Institutes of Health Stroke Scale; PT, prothrombin time; SMD, standardised mean difference; TIA, transient ischaemic attack (without residual deficits).

^a^ All treatments listed were registered prior to thrombolysis.

Following 1:1 propensity score matching, there were 766 patients with bridging thrombolysis and 766 patients with EVT only, and the cohorts were well balanced on all included characteristics (all SMDs < 0.1; Table 1).

### Risk of ICH with bridging thrombolysis versus EVT alone at 30 and 90 days

**FIGURE 2:**
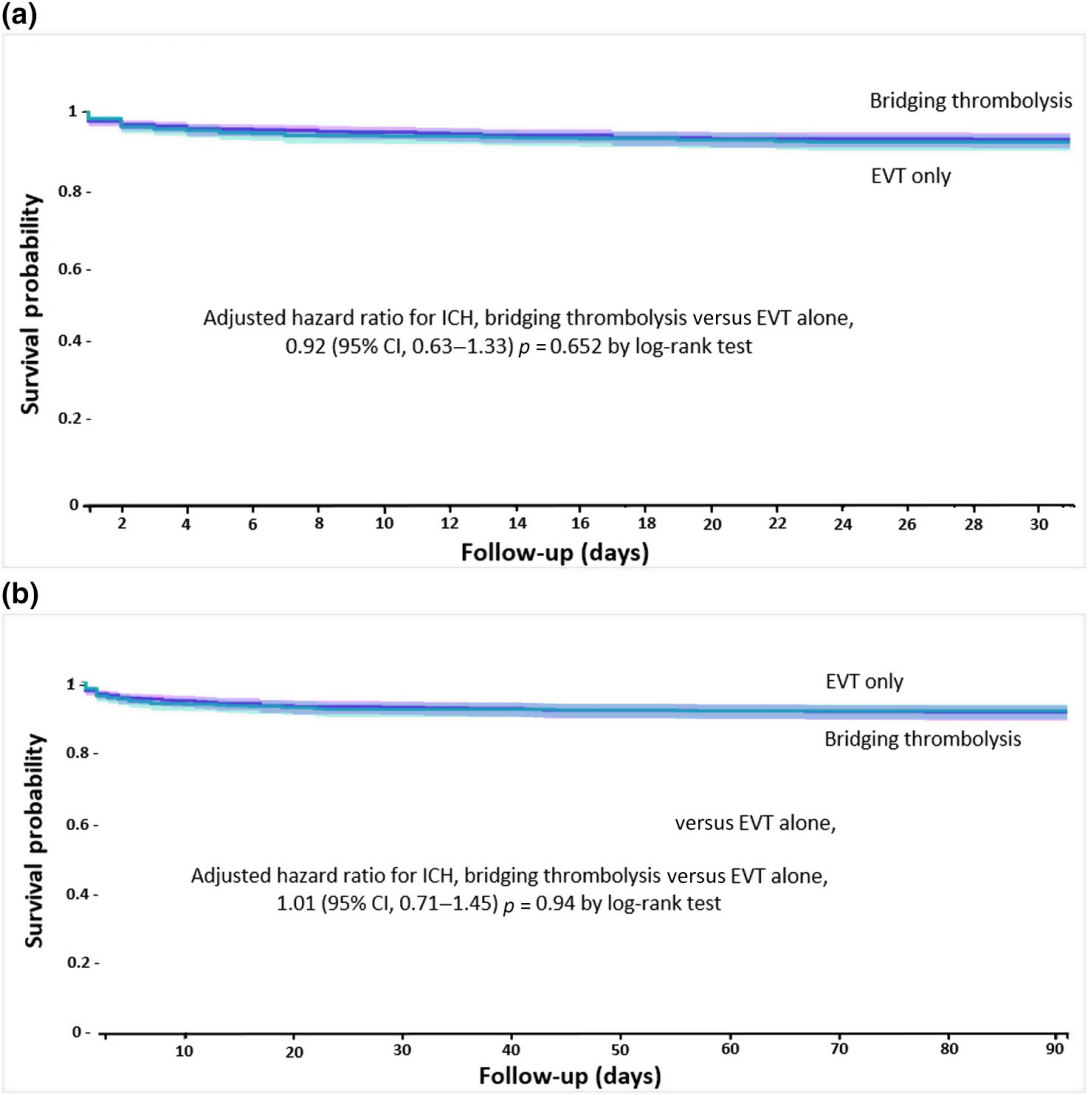
(a) Kaplan–Meier curve depicting the risk of intracerebral haemorrhage (ICH) events within 30 days after bridging thrombolysis in patients with ischaemic stroke compared to endovascular thrombectomy (EVT) alone. (b) Kaplan–Meier curve depicting the risk of ICH events within 90 days after bridging thrombolysis in patients with ischaemic stroke compared to EVT alone. CI, confidence interval.

Following propensity score matching, Kaplan–Meier survival analyses showed a similar risk of 30‐ and 90‐day ICH for anticoagulated patients with bridging thrombolysis compared to propensity score‐matched EVT‐only patients (adjusted HR [aHR] = 0.92, 95% CI = 0.63–1.33, survival probability 92.6% vs. 92.1%, log‐rank test *p* = 0.65; and HR = 1.01, 95% CI = 0.71–1.45, survival probability 91.2% vs. 91.7%, log‐rank test *p* = 0.94, respectively; Figure 2a,b).

### Risk of SAH with bridging thrombolysis versus EVT alone at 30 and 90 days

**FIGURE 3:**
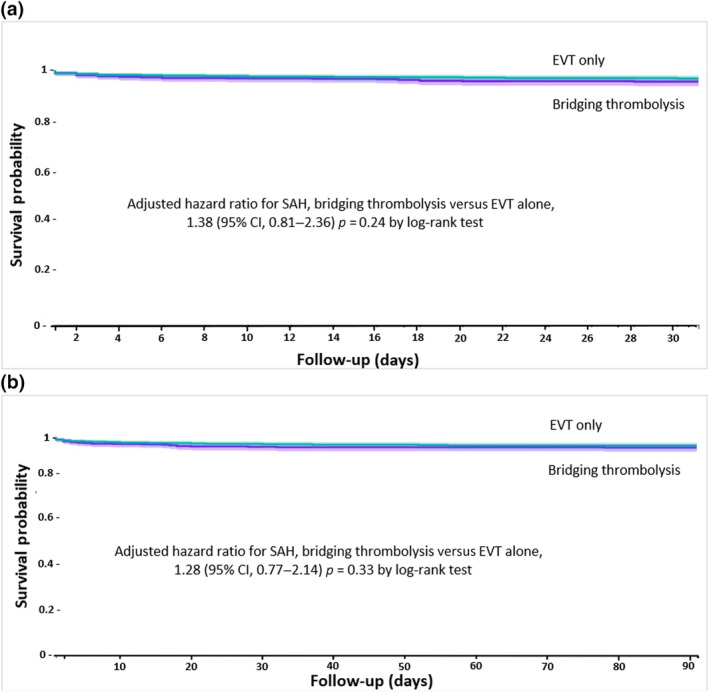
(a) Kaplan–Meier curve depicting the risk of subarachnoid haemorrhage (SAH) events within 30 days after bridging thrombolysis in patients with ischaemic stroke compared to endovascular thrombectomy (EVT) alone. (b) Kaplan–Meier curve depicting the risk of SAH events within 90 days after bridging thrombolysis in patients with ischaemic stroke compared to EVT alone. CI, confidence interval.

There was a similar risk of 30‐ and 90‐day SAH for anticoagulated patients with bridging thrombolysis compared to propensity score‐matched EVT‐only patients (aHR = 1.38, 95% CI = 0.81–2.36, survival probability 95.5 vs. 96.7%, log‐rank test *p* = 0.24; and HR = 1.28, 95% CI = 0.77–2.14, survival probability 95.1% vs. 96.1%, log‐rank test *p* = 0.33, respectively; Figure 3a,b).

### Risk of death with bridging thrombolysis versus EVT alone at 30 and 90 days

**FIGURE 4:**
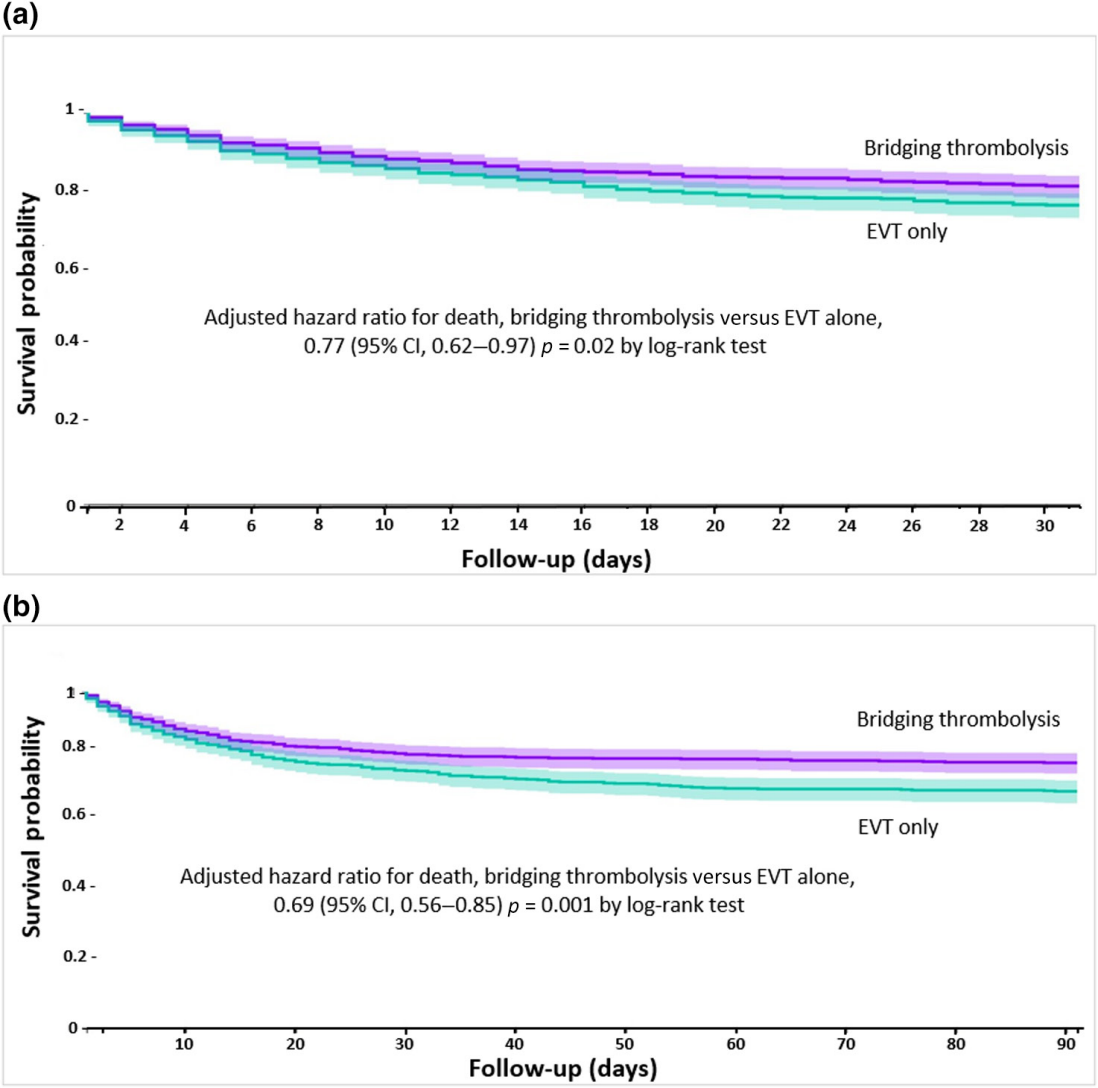
(a) Kaplan–Meier curve depicting the risk of subarachnoid haemorrhage events within 30 days after bridging thrombolysis in patients with ischaemic stroke compared to endovascular thrombectomy (EVT) alone. (b) Kaplan–Meier curve depicting the risk of subarachnoid haemorrhage events within 90 days after bridging thrombolysis in patients with ischaemic stroke compared to EVT alone. CI, confidence interval.

Nevertheless, there was a significantly lower risk for death at 30 and 90 days in bridging thrombolysis patients compared to propensity score‐matched EVT‐only patients (HR = 0.77, 95% CI = 0.62–0.97, survival probability 81.3 vs. 76.4%, log‐rank test *p* = 0.02; and HR = 0.69, 95% CI = 0.56–0.85, survival probability 78.8% vs. 70.3%, log‐rank test *p* = 0.001, respectively; Figure 4a,b).

## DISCUSSION

In this study, our principal finding was that anticoagulated patients with ischaemic stroke and AF receiving bridging thrombolysis was associated with lower all‐cause mortality within 30 and 90 days compared to those treated with EVT alone. No significant difference in risk of ICH and SAH was observed between the two groups.

AF plays a pivotal role in the incidence of large vessel occlusion, compelling the need for EVT treatment. One multicentre observational study of 2941 patients who underwent EVT for anterior LVO included 1347 (45.8%) patients with AF. In this subgroup, bridging thrombolysis was significantly associated with better function at 90 days and lower mortality, but not significantly associated with sICH, compared to EVT alone [10]. Notably, prior anticoagulation with a vitamin K antagonist (VKA) was linked to an increased risk of sICH compared to DOACs [10]. However, 73.4% of the AF cohort were not on anticoagulants, and only a very limited proportion in the bridging group were on DOACs or VKAs (1% and 8.3%, respectively). This limits the generalisability of these results to anticoagulated patients with AF. In contrast, our study supports previous findings that bridging thrombolysis does not influence ICH rates while reducing all‐cause mortality within 30 and 90 days in anticoagulated ischaemic stroke patients with AF, as opposed to EVT alone.

Additionally, a retrospective analysis of the multicentre IAC (Initiation of Anticoagulation in Cardioembolic Stroke) study, involving 522 AF patients who underwent EVT for anterior and posterior LVO, revealed that that bridging thrombolysis was associated with fewer EVT device passes, reduced 90‐day mortality, and comparable rates of successful recanalisation and haemorrhagic transformation compared to EVT alone [15]. Further analysis stratified by anticoagulant type showed that warfarin was significantly associated with higher risk of haemorrhagic transformation, whereas DOACs had similar rates of haemorrhagic transformation and mortality at 90 days between bridging and EVT‐alone groups [15].

There is some debate whether anticoagulation per se increases the risk of ICH or mortality for ischaemic stroke patients undergoing bridging thrombolysis compared to EVT alone. A retrospective multicentre international study from four countries (Australia, Canada, China, and India), including 563 patients with AF who underwent EVT, demonstrated that bridging thrombolysis in nonanticoagulated patients resulted in better functional outcomes, but similar mortality and sICH rates compared to EVT alone in nonanticoagulated patients [16]. However, there was a nonsignificant trend towards better functional outcomes, higher successful recanalisation, lower mortality, and similar ICH rates with bridging thrombolysis with anticoagulants compared to those without anticoagulants. The small sample size in bridging thrombolysis groups (anticoagulants *n* = 22 [3.90%] vs. no anticoagulants *n* = 212 [37.6%]) may affect the robustness of these results. In contrast to our study, this analysis did not compare bridging thrombolysis to EVT alone in anticoagulated patients with AF.

In a large thrombectomy registry involving 6461 patients who underwent EVT for anterior and posterior LVO, a subgroup analysis of 2311 (35.8%) AF patients receiving bridging thrombolysis showed increased haemorrhagic complications with no difference in functional outcomes or successful recanalisation, and lower mortality compared to AF patients treated with EVT alone [8]. The increased haemorrhagic complications were marginally significant in the bridging thrombolysis group compared to EVT alone (9.2% vs. 6.9%, *p* < 0.0477).

Furthermore, a subgroup analysis of the MR CLEAN study, which included 327 AF patients who underwent EVT for anterior LVO, found that bridging thrombolysis was not significantly associated with 90‐day good functional outcomes, successful reperfusion, or mortality, compared to EVT alone [9]. However, there was a higher rate of ICH in the bridging group compared to EVT alone, but this difference was not statistically significant (5.9% vs. 2.8%, adjusted odds ratio = 2.18, 95% CI = 0.60–7.91). In addition, a retrospective multicentre international study from six countries (Singapore, China, Taiwan, Germany, United Kingdom, and Italy), assessing the effects of bridging thrombolysis and EVT alone in anterior circulation LVO patients with AF compared to patients without AF, found no difference in good functional outcomes, sICH, successful recanalisation, and in‐hospital mortality, in those treated with bridging thrombolysis versus EVT alone [17]. However, this study might be limited by its heterogenous populations and relatively small sample size. Furthermore, previous meta‐analysis of randomised clinical trials demonstrated lower odds of ICH in patients with ischaemic stroke undergoing IVT who had recent DOACs compared to patients with no DOACs [11]. Of note, AF patients on oral anticoagulants (OACs) have smaller infarct volumes and lower NIHSS, which is a plausible explanation [18].

Although IVT should not be omitted in patients with LVO planning to undergo EVT, patients on OACs are usually not offered this treatment option. The present study provides evidence that bridging IVT might be beneficial even in AF patients on OACs, with no increased harm from ICH or SAH at 30 and 90 days. However, a powered randomised clinical trial that examines the effects of bridging thrombolysis compared to EVT alone in anticoagulated patients with acute ischaemic stroke and AF on various clinical outcomes is needed.

The low percentage of anticoagulated AF patients is noteworthy; however, it is important to consider the clinical scenarios that might necessitate the use of oral anticoagulation. These include a high risk of thromboembolic events, recent initiation of anticoagulants, and transition between different anticoagulation therapies. Such clinical decisions are made to balance the individual risks and benefits for each patient.

### Strengths and limitations

This study has several strengths. First, it uses propensity score matching to control for clinically and prognostically relevant factors, thus minimising the risk of bias from confounding. Second, despite the use of ICD‐10 codes, the study expands upon the existing literature by providing additional consistent findings using real‐world patient data from various health care organizations and regions. This approach provides a broader representation of patient demographics and outcomes. Also, due to the de‐identified dataset, we cannot validate the ICD‐10 codes used in the TriNetX network. Lastly, propensity score matching can reduce selection bias in observational studies by creating a more balanced comparison between treatment and control groups, thus mimicking the characteristics of a randomised controlled trial. This could enhance the validity of causal inferences drawn from the study [19].

This study has several limitations. The ICD‐10 codes that were extracted from the electronic medical records are inherently restricted and may show variability across health care organisations, and the impact of attending different health care organisations cannot be ascertained due to confidentiality considerations. TriNetX provides real‐time access to anonymised electronic health care records from more than 110 health care organisations worldwide, predominantly in the United States. To adhere to legal and ethical standards and to prevent the re‐identification of data, the identities of participating organisations and their specific data contributions remain undisclosed. Within the TriNetX platform, no patient‐identifiable information is accessible; only de‐identified data are presented through aggregated counts and statistical summaries [20]. Selection of patients is based on ICD‐10 codes, and there is potential for misclassification inherent in all electronic health record analyses; however, previous studies have shown ICD‐10 codes have high accuracy for detecting AF [21, 22]. The variability in the intensity and timing of anticoagulant re‐initiation following an index ischaemic stroke could affect the rates of ICH and death in both groups. Our analysis was limited in its ability to compare the risk of ICH between VKAs and DOACs due to the small numbers of patients receiving these medications. Given the size of the cohort after propensity score matching, further stratifications were not performed due to limited statistical power, but future studies in larger cohorts could consider the impact of type of AF, age, and sex. Furthermore, the stroke etiology in AF patients might be attributable to AF itself or other atherosclerotic artery occlusions. The specific cause of death was not available for analysis, leading to a categorisation of death as “all‐cause mortality.” Additionally, deaths occurring outside the TriNetX network were not captured. Although the study spanned more than 30 multi‐health care organisation centres, its findings may not be fully generalisable to the broader US population or other populations. The analysis was propensity score matched for factors such as age, sex, stroke severity, and other comorbidities; however, the variables collected in the electronic database were not pre‐determined, and unmeasured residual prognostic confounding may have influenced some of our results, such as time to thrombolysis metrics (symptom onset to successful recanalisation, time “door‐in to door‐out”), successful recanalisation, prestroke disability (modified Rankin Scale [mRS] score = 4–6), and good functional status (mRS score = 0–2). There were also limited data on comparisons of ethnic differences, given differences in bleeding and stroke in some ethnicities [23, 24]. Lastly, the study lacked comprehensive data on the NIHSS score for evaluating both prestroke and post‐EVT functional status (mRS).

## CONCLUSIONS

In anticoagulated patients with acute ischaemic stroke and AF, bridging thrombolysis was not associated with increased risk of ICH or SAH, but there was a significantly lower risk of all‐cause mortality at 30‐ and 90‐day follow‐up, compared with EVT alone.

## FUNDING INFORMATION

M.A. is funded by a PhD studentship from King Saud University.

## CONFLICT OF INTEREST STATEMENT

S.L.H. has received grant funding from Bristol Myers Squibb (BMS) outside of the submitted work. D.A.L. has received investigator‐initiated educational grants from BMS, has been a speaker for Bayer, Boehringer Ingelheim, and BMS/Pfizer, and has consulted for BMS and Boehringer Ingelheim, all outside the submitted work. P.A. is an employee of TriNetX. G.Y.H.L. has been a consultant and speaker for BMS/Pfizer, Boehringer Ingelheim, and Daiichi‐Sankyo; no fees are received personally. None of the other authors has any conflict of interest to disclose.

## ETHICS STATEMENT

Ethical approval for this study was waived by TriNetX. Research studies using the TriNetX federated network do not require ethical approval or patient‐informed consent, as no patient‐identifiable information is received.

## Supporting information


TABLES S1–S2.

## Data Availability

The data underlying this article are available in the TriNetX research network at https://live.trinetx.com with a request for access to the TriNetX network, but costs may be incurred.

## References

[1] Powers WJ , Rabinstein AA , Ackerson T , et al. Guidelines for the early management of patients with acute ischemic stroke: 2019 update to the 2018 guidelines for the early management of acute ischemic stroke: a guideline for healthcare professionals from the American Heart Association/American Stroke Association. Stroke. 2019;50:e344‐418. 31662037 10.1161/STR.0000000000000211

[2] Turc G , Bhogal P , Fischer U , et al. European stroke organisation (ESO)‐European Society for Minimally Invasive Neurological Therapy (ESMINT) guidelines on mechanical thrombectomy in acute ischemic stroke. J NeuroInterventional Surg. 2023;15:e8. 10.1136/neurintsurg-2018-014569 30808653

[3] Majoie CB , Cavalcante F , Gralla J , et al. Value of intravenous thrombolysis in endovascular treatment for large‐vessel anterior circulation stroke: individual participant data meta‐analysis of six randomised trials. Lancet. 2023;402:965‐974. 37640037 10.1016/S0140-6736(23)01142-X

[4] Turc G , Tsivgoulis G , Audebert HJ , et al. European stroke organisation (ESO)–European Society for Minimally Invasive Neurological Therapy (ESMINT) expedited recommendation on indication for intravenous thrombolysis before mechanical thrombectomy in patients with acute ischemic stroke and anterior circulation large vessel occlusion. J NeuroInterventional Surg. 2022;14:209‐227. 10.1136/neurintsurg-2021-018589 35115395

[5] Henninger N , Goddeau RP Jr , Karmarkar A , Helenius J , McManus DD . Atrial fibrillation is associated with a worse 90‐day outcome than other cardioembolic stroke subtypes. Stroke. 2016;47:1486‐1492. 27217503 10.1161/STROKEAHA.116.012865 PMC4880452

[6] Šaňák D , Herzig R , Král M , et al. Is atrial fibrillation associated with poor outcome after thrombolysis? J Neurol. 2010;257:999‐1003. 20127250 10.1007/s00415-010-5452-4

[7] Berge E , Whiteley W , Audebert H , et al. European stroke organisation (ESO) guidelines on intravenous thrombolysis for acute ischaemic stroke. Eur Stroke J. 2021;6:I‐LXII. 10.1177/2396987321989865 PMC7995316 33817340

[8] Akbik F , Alawieh A , Dimisko L , et al. Bridging thrombolysis in atrial fibrillation stroke is associated with increased hemorrhagic complications without improved outcomes. J NeuroInterv Surg. 2022;14:979‐984. 34819345 10.1136/neurintsurg-2021-017954

[9] Chalos V , LeCouffe NE , Uyttenboogaart M , et al. Endovascular treatment with or without prior intravenous alteplase for acute ischemic stroke. J Am Heart Assoc. 2019;8:e011592. 31140355 10.1161/JAHA.118.011592 PMC6585366

[10] Mujanovic A , Kurmann CC , Dobrocky T , et al. Bridging intravenous thrombolysis in patients with atrial fibrillation. Front Neurol. 2022;13:945338. 35989924 10.3389/fneur.2022.945338 PMC9382124

[11] Meinel TR , Wilson D , Gensicke H , et al. Intravenous thrombolysis in patients with ischemic stroke and recent ingestion of direct Oral anticoagulants. JAMA Neurol. 2023;80:233‐243. 36807495 10.1001/jamaneurol.2022.4782 PMC9857462

[12] Patel J , Bhaskar SM . Atrial fibrillation and reperfusion therapy in acute Ischaemic stroke patients: prevalence and outcomes—a comprehensive systematic review and meta‐analysis. Neurol Int. 2023;15:1014‐1043. 37755356 10.3390/neurolint15030065 PMC10537209

[13] Powers WJ , Rabinstein AA , Ackerson T , et al. 2018 guidelines for the early management of patients with acute ischemic stroke: a guideline for healthcare professionals from the American Heart Association/American Stroke Association. Stroke. 2018;49:e46‐e99. 29367334 10.1161/STR.0000000000000158

[14] Lees KR , Bath PM , Schellinger PD , et al. Contemporary outcome measures in acute stroke research: choice of primary outcome measure. Stroke. 2012;43:1163‐1170. 22426313 10.1161/STROKEAHA.111.641423

[15] Yaghi S , Mistry E , de Havenon A , et al. Effect of alteplase use on outcomes in patients with atrial fibrillation: analysis of the initiation of anticoagulation after cardioembolic stroke study. J Am Heart Assoc. 2021;10:e020945. 34323120 10.1161/JAHA.121.020945 PMC8475683

[16] Lin L , Blair C , Fu J , et al. Prior anticoagulation and bridging thrombolysis improve outcomes in patients with atrial fibrillation undergoing endovascular thrombectomy for anterior circulation stroke. J NeuroInterv Surg. 2023;15:e433‐e437. 36944493 10.1136/jnis-2022-019560

[17] Loo JH , Leow AS , Jing M , et al. Impact of atrial fibrillation on the treatment effect of bridging thrombolysis in ischemic stroke patients undergoing endovascular thrombectomy: a multicenter international cohort study. J NeuroInterv Surg. 2023;15:1274‐1279. 36609541 10.1136/jnis-2022-019590

[18] Meinel TR , Branca M , De Marchis GM , et al. Prior anticoagulation in patients with ischemic stroke and atrial fibrillation. Ann Neurol. 2021;89:42‐53. 32996627 10.1002/ana.25917 PMC7756294

[19] Austin PC . An introduction to propensity score methods for reducing the effects of confounding in observational studies. Multivar Behav Res. 2011;46:399‐424. 10.1080/00273171.2011.568786 PMC3144483 21818162

[20] Palchuk MB , London JW , Perez‐Rey D , et al. A global federated real‐world data and analytics platform for research. JAMIA Open. 2023;6:ooad035. 37193038 10.1093/jamiaopen/ooad035 PMC10182857

[21] Norberg J , Backstrom S , Jansson JH , Johansson L . Estimating the prevalence of atrial fibrillation in a general population using validated electronic health data. Clin Epidemiol. 2013;5:475‐481. 24353441 10.2147/CLEP.S53420 PMC3862395

[22] Cozzolino F , Montedori A , Abraha I , et al. A diagnostic accuracy study validating cardiovascular ICD‐9‐CM codes in healthcare administrative databases. The Umbria data‐value project. PLoS One. 2019;14:e0218919. 31283787 10.1371/journal.pone.0218919 PMC6613689

[23] Gorog DA , Gue YX , Chao TF , et al. Assessment and mitigation of bleeding risk in atrial fibrillation and venous thromboembolism: executive summary of a European and Asia‐Pacific expert consensus paper. Thromb Haemost. 2022;122:1625‐1652. 35793691 10.1055/s-0042-1750385

[24] Kim HK , Tantry US , Smith SC Jr , et al. The east Asian paradox: an updated position statement on the challenges to the current antithrombotic strategy in patients with cardiovascular disease. Thromb Haemost. 2021;121:422‐432. 33171520 10.1055/s-0040-1718729

