# Supplementary material for: Impact of bridging thrombolysis versus endovascular thrombectomy alone on outcomes in anticoagulated patients with atrial fibrillation and acute ischaemic stroke
Source: Eur J Neurol. 2024 Aug 23;31(12):e16453. doi: 10.1111/ene.16453 (PMC11554861; doi:10.1111/ene.16453)
Supplement: Supplementary file 1 — TABLES S1–S2. [file ENE-31-e16453-s001.docx]

**Supplementary Table 1**: ICD-10-CM or CPT codes for atrial fibrillation patients with acute ischaemic stroke who treated by bridging thrombolysis or EVT alone.

| **ICD-10 and CPT codes** | |
| --- | --- |
| **ICD-10 code** | **Description** |
| I63.52  I63.53 | Cerebral infarction (thrombosis & embolism & occlusion or stenosis of precerebral, anterior, middle, carotid, basilar and vertebral) |
| I48 | Atrial fibrillation and flutter |
| BL110 | Anticoagulants |
| 259280 | Tenecteplase |
| 8410 | Alteplase |
| **ICD-10 code** | **Stroke severity** |
| R29.7 | National Institutes of Health Stroke Scale (NIHSS) score |
| **CPT** | **Mechanical Endovascular Reperfusion Procedures** |
| 03CG3Z7 | Extirpation of Matter from Intracranial Artery using Stent Retriever, Percutaneous Approach |
| 03CG3ZZ | Extirpation of Matter from Intracranial Artery, Percutaneous Approach |
| 03CG4ZZ | Extirpation of Matter from Intracranial Artery, Percutaneous Endoscopic Approach |
| 03CH3Z7 | Extirpation of Matter from Right Common Carotid Artery using Stent Retriever, Percutaneous Approach |
| 03CH3ZZ | Extirpation of Matter from Right Common Carotid Artery, Percutaneous Approach |
| 03CH4ZZ | Extirpation of Matter from Right Common Carotid Artery, Percutaneous Endoscopic Approach |
| 03CJ3Z7 | Extirpation of Matter from Left Common Carotid Artery using Stent Retriever, Percutaneous Approach |
| 03CJ3ZZ | Extirpation of Matter from Left Common Carotid Artery, Percutaneous Approach |
| 03CJ4ZZ | Extirpation of Matter from Left Common Carotid Artery, Percutaneous Endoscopic Approach |
| 03CK3Z7 | Extirpation of Matter from Right Internal Carotid Artery using Stent Retriever, Percutaneous Approach |
| 03CK3ZZ | Extirpation of Matter from Right Internal Carotid Artery, Percutaneous Approach |
| 03CK4ZZ | Extirpation of Matter from Right Internal Carotid Artery, Percutaneous Endoscopic Approach |
| 03CL3Z7 | Extirpation of Matter from Left Internal Carotid Artery using Stent Retriever, Percutaneous Approach |
| 03CL3ZZ | Extirpation of Matter from Left Internal Carotid Artery, Percutaneous Approach |
| 03CL4ZZ | Extirpation of Matter from Left Internal Carotid Artery, Percutaneous Endoscopic Approach |
| 03CP3Z7 | Extirpation of Matter from Right Vertebral Artery using Stent Retriever, Percutaneous Approach |
| 03CP3ZZ | Extirpation of Matter from Right Vertebral Artery, Percutaneous Approach |
| 03CP4ZZ | Extirpation of Matter from Right Vertebral Artery, Percutaneous Endoscopic Approach |
| 03CQ3Z7 | Extirpation of Matter from Left Vertebral Artery using Stent Retriever, Percutaneous Approach |
| 03CQ3ZZ | Extirpation of Matter from Left Vertebral Artery, Percutaneous Approach |
| 03CQ4ZZ | Extirpation of Matter from Left Vertebral Artery, Percutaneous Endoscopic Approach |
| **Thrombectomy Root Procedures** | |
| 037G3ZZ | Dilation of Intracranial Artery, Percutaneous Approach |
| 037G4ZZ | Dilation of Intracranial Artery, Percutaneous Endoscopic Approach |
| 037H3ZZ | Dilation of Right Common Carotid Artery, Percutaneous Approach |
| 037H4ZZ | Dilation of Right Common Carotid Artery, Percutaneous Endoscopic Approach |
| 037J3ZZ | Dilation of Left Common Carotid Artery, Percutaneous Approach |
| 037J4ZZ | Dilation of Left Common Carotid Artery, Percutaneous Endoscopic Approach |
| 037K3ZZ | Dilation of Right Internal Carotid Artery, Percutaneous Approach |
| 037K4ZZ | Dilation of Right Internal Carotid Artery, Percutaneous Endoscopic Approach |
| 037L3ZZ | Dilation of Left Internal Carotid Artery, Percutaneous Approach |
| 037L4ZZ | Dilation of Left Internal Carotid Artery, Percutaneous Endoscopic Approach |
| 037P3ZZ | Dilation of Right Vertebral Artery, Percutaneous Approach |
| 037P4ZZ | Dilation of Right Vertebral Artery, Percutaneous Endoscopic Approach |
| 037Q3ZZ | Dilation of Left Vertebral Artery, Percutaneous Approach |
| 037Q4ZZ | Dilation of Left Vertebral Artery, Percutaneous Endoscopic Approach |
| **Intracerebral Haemorrhage** | |
| I61.0 | Nontraumatic intracerebral hemorrhage in hemisphere, subcortical |
| I61.1 | Nontraumatic intracerebral hemorrhage in hemisphere, cortical |
| I61.2 | Nontraumatic intracerebral hemorrhage in hemisphere, unspecified |
| I61.3 | Nontraumatic intracerebral hemorrhage in brain stem |
| I61.4 | Nontraumatic intracerebral hemorrhage in cerebellum |
| I61.5 | Nontraumatic intracerebral hemorrhage, intraventricular |
| I616 | Nontraumatic intracerebral hemorrhage, multiple localized |
| I618 | Other nontraumatic intracerebral hemorrhage |
| I619 | Nontraumatic intracerebral hemorrhage, unspecified |
| **Subarachnoid haemorrhage** | |
| 160.1 | Nontraumatic subarachnoid hemorrhage from middle cerebral |
| 160.2 | Nontraumatic subarachnoid hemorrhage from anterior communicating artery |
| 160.3 | Nontraumatic subarachnoid hemorrhage from posterior communicating artery |
| 160.4 | Nontraumatic subarachnoid hemorrhage from basilar artery |
| 160.5 | Nontraumatic subarachnoid hemorrhage from vertebral artery |
| 160.6 | Nontraumatic subarachnoid hemorrhage from other intracranial arteries |
| 160.8 | Other nontraumatic subarachnoid hemorrhage |
| 160.9 | Nontraumatic subarachnoid hemorrhage, unspecified |

ICD-10-CM: International Classification of Diseases-10^th^ Revision-Clinical Modification, CPT: Current Procedural Terminology.

**Supplementary Table 2:** STROBE Statement—Checklist of items that should be included in reports of cohort studies.

|  | Item No | Recommendation | **Page/section information** |
| --- | --- | --- | --- |
| **Title and abstract** | 1 | (*a*) Indicate the study’s design with a commonly used term in the title or the abstract | Page 1-3; title page and abstract |
|  |  | (*b*) Provide in the abstract an informative and balanced summary of what was done and what was found | Page 1-3; title page and abstract |
| Introduction | | |  |
| Background/rationale | 2 | Explain the scientific background and rationale for the investigation being reported | Page 4-5; introduction |
| Objectives | 3 | State specific objectives, including any prespecified hypotheses | Page 5; introduction |
| Methods | | |  |
| Study design | 4 | Present key elements of study design early in the paper | Page 6; methods |
| Setting | 5 | Describe the setting, locations, and relevant dates, including periods of recruitment, exposure, follow-up, and data collection | Page 6-7; methods |
| Participants | 6 | (*a*) Give the eligibility criteria, and the sources and methods of selection of participants. Describe methods of follow-up | Page 6-7; methods |
|  |  | (*b*) For matched studies, give matching criteria and number of exposed and unexposed | Page 6; methods + figure 1 |
| Variables | 7 | Clearly define all outcomes, exposures, predictors, potential confounders, and effect modifiers. Give diagnostic criteria, if applicable | Page 7; methods + table 1 + table S1 |
| Data sources/ measurement | 8* | For each variable of interest, give sources of data and details of methods of assessment (measurement). Describe comparability of assessment methods if there is more than one group | Page 6-7; methods |
| Bias | 9 | Describe any efforts to address potential sources of bias | Page 6-7; methods |
| Study size | 10 | Explain how the study size was arrived at | Page 6-7; methods + figure 1 |
| Quantitative variables | 11 | Explain how quantitative variables were handled in the analyses. If applicable, describe which groupings were chosen and why | Page 6-7; methods |
| Statistical methods | 12 | (*a*) Describe all statistical methods, including those used to control for confounding | Page 7-8; methods |
|  |  | (*b*) Describe any methods used to examine subgroups and interactions | N/A |
|  |  | (*c*) Explain how missing data were addressed | N/A |
|  |  | (*d*) If applicable, explain how loss to follow-up was addressed | N/A |
|  |  | (*e*) Describe any sensitivity analyses | N/A |
| Results | | |  |
| Participants | 13* | (a) Report numbers of individuals at each stage of study—eg numbers potentially eligible, examined for eligibility, confirmed eligible, included in the study, completing follow-up, and analysed | Page 8-9; results |
|  |  | (b) Give reasons for non-participation at each stage | N/A |
|  |  | (c) Consider use of a flow diagram | Page 8; results + figure 1 |
| Descriptive data | 14* | (a) Give characteristics of study participants (eg demographic, clinical, social) and information on exposures and potential confounders | Page 8-9; results + table 1 |
|  |  | (b) Indicate number of participants with missing data for each variable of interest | N/A |
|  |  | (c) Summarise follow-up time (eg, average and total amount) | Page 8-9; results |
| Outcome data | 15* | Report numbers of outcome events or summary measures over time |  |
| Main results | 16 | (*a*) Give unadjusted estimates and, if applicable, confounder-adjusted estimates and their precision (eg, 95% confidence interval). Make clear which confounders were adjusted for and why they were included | Page 8; results + Table 1 + Figure 2&3&4 |
|  |  | (*b*) Report category boundaries when continuous variables were categorized | N/A |
|  |  | (*c*) If relevant, consider translating estimates of relative risk into absolute risk for a meaningful time period | Page 8; results + table 1 |
| Other analyses | 17 | Report other analyses done—eg analyses of subgroups and interactions, and sensitivity analyses | N/A |
| Discussion | | |  |
| Key results | 18 | Summarise key results with reference to study objectives | Page 10-12; discussion |
| Limitations | 19 | Discuss limitations of the study, taking into account sources of potential bias or imprecision. Discuss both direction and magnitude of any potential bias | Page 12-13; limitations |
| Interpretation | 20 | Give a cautious overall interpretation of results considering objectives, limitations, multiplicity of analyses, results from similar studies, and other relevant evidence | Page 10-14; limitations + conclusion |
| Generalisability | 21 | Discuss the generalisability (external validity) of the study results | Page 10-14; limitations + conclusion |
| Other information | | |  |
| Funding | 22 | Give the source of funding and the role of the funders for the present study and, if applicable, for the original study on which the present article is based | Page 15 |

*Give information separately for exposed and unexposed groups.

**Note:** An Explanation and Elaboration article discusses each checklist item and gives methodological background and published examples of transparent reporting. The STROBE checklist is best used in conjunction with this article (freely available on the Web sites of PLoS Medicine at http://www.plosmedicine.org/, Annals of Internal Medicine at http://www.annals.org/, and Epidemiology at http://www.epidem.com/). Information on the STROBE Initiative is available at http://www.strobe-statement.org.
